# Protective Effects of Methane-Rich Saline on Rats with Lipopolysaccharide-Induced Acute Lung Injury

**DOI:** 10.1155/2017/7430193

**Published:** 2017-05-02

**Authors:** Aijun Sun, Weiheng Wang, Xiaojian Ye, Yang Wang, Xiangqun Yang, Zhouheng Ye, Xuejun Sun, Chuansen Zhang

**Affiliations:** ^1^Institute of Biomedical Engineering, Second Military Medical University, Shanghai 200433, China; ^2^Department of Orthopaedics, Changzheng Hospital Affiliated to the Second Military Medical University, Shanghai 200003, China; ^3^Department of Navy Aeromedicine, Second Military Medical University, Shanghai 200433, China

## Abstract

*Objective.* The aim of this research is to evaluate the protective effects of methane-rich saline (MS) on lipopolysaccharide- (LPS-) induced acute lung injury (ALI) and investigate its potential antioxidative, anti-inflammatory, and antiapoptotic activities. *Methods.* LPS-induced (20 mg/kg) ALI rats were injected with MS (2 ml/kg and 20 ml/kg) before the initiation of LPS induction. Survival rate was determined until 96 h after LPS was induced. Lung injury was assayed by oxygenation index, lung permeability index (LPI), wet-to-dry weight (W/D), and histology. The cells in the bronchoalveolar lavage fluid (BALF) were counted. Oxidative stress was examined by the level of malondialdehyde (MDA) and superoxide dismutase (SOD). Inflammatory factors including tumor necrosis factor-*α* (TNF-*α*), interleukin-1*β* (IL-1*β*), and interleukin-6 (IL-6) in BALF were determined by ELISA. Lung tissue apoptosis was detected by TUNEL staining and western blotting of caspase-3. *Results.* It was found that methane significantly prolonged the rat survival, decreased the lung W/D ratio and the content of the inflammatory factors, and reduced the amount of caspase-3 and apoptotic index. In addition, MS increased the level of SOD and decreased the level of MDA significantly. *Conclusions.* MS protects the LPS-challenged ALI via antioxidative, anti-inflammatory, and antiapoptotic effect, which may prove to be a novel therapy for the clinical management of ALI.

## 1. Introduction

Acute lung injury (ALI) and the acute respiratory distress syndrome (ARDS) are commonly encountered in severe diseases such as shock, burn, and infection and are considered as the major causes of acute respiratory failure, seriously threatening human health [1]. According to the latest statistics, the morbidity and mortality of patients in intensive care units (ICUs) are 20 and 50%, respectively, depending on different areas [2, 3]. Despite intensive investigations in animals and patients, the etiology and molecular mechanisms of ALI/ARDS have not been fully clarified. ALI/ARDS are clinically characterized by progressive hypoxemia and respiratory distress syndrome, contributing to diffuse pulmonary interstitial and alveolar edema caused by pulmonary capillary endothelial cells and alveolar epithelial cell injury [4]. Recent evidence suggests that the main pathogenesis of ALI includes cytokine imbalance, oxidative stress, and apoptosis [5]. Cytokine imbalance means the imbalance of proinflammatory cytokines and anti-inflammatory cytokines in the lung tissue in the presence of ALI. As a result, large numbers of activated mononuclear macrophages released large amounts of proinflammatory cytokines, leading to lung and systemic inflammatory responses. In the pathogenesis of ALI, activated neutrophils are produced, releasing large amounts of reactive oxygen species (ROS), which directly damage lung parenchymal cells through the cell membrane lipid oxidation. ROS can damage the capillary basement membrane and other stromal components at the same time. The combined action of inflammation and oxidative stress is the main factor for the increased permeability of capillary pressure, leading to tissue edema and more severe tissue injury. ALI-associated apoptosis of lung cells is significantly increased. Lung vascular endothelial cells begin appearing apoptotic in the early phase of ALI. In the recovery period, apoptosis is also involved in lung reconstruction and recovery of respiratory function.

Current treatment strategies to ALI/ARDS address the primary disease, respiratory support therapy, and fluid management. Although the medical and ventilator support therapies have improved during recent decades, the effect remains limited with many side effects, and the morbidity and mortality of ALI/ARDS remain high [6, 7]. Thus, it is imperative to develop effective strategies for the treatment of ALI without significant or with only mild side effects. Some recent studies [8–10] found that hydrogen-rich saline could exert a potential effect against inflammation and oxidative stress-associated pathologies in ALI, which provides a novel strategy for the treatment of ALI. However, because methanogens in the intestine can partially produce hydrogen and convert hydrogen to methane in vivo [11, 12], there are controversies over the therapeutic effect of hydrogen and methane and the effect of methane on ALI is unclear in vivo. Methane is a small organic-reducing molecule of the simplest alkane. Inhalation of large amounts of methane can cause lung ischemia injury because of relative oxygen deficiency [13]. Recent studies have demonstrated that methane is a new biologically active molecule with antioxidative, anti-inflammatory, and antiapoptotic activities [14, 15]. Our previous studies [16, 17] also demonstrated that methane protected the liver against ischemia/reperfusion (I/R) injury and the myocardium against myocardial infarction through its antioxidative, anti-inflammatory, and antiapoptotic activities.

However, the therapeutic effect of methane on ALI is unclear. This study intends to examine the therapeutic effect of methane in a rat model of ALI induced by LPS and explore the underlying specific mechanisms.

## 2. Materials and Methods

### 2.1. Animals

The adult male Sprague-Dawley (SD) rats weighing 200–220 g (Experimental Animal Center of the Second Military Medical University, Shanghai, China) were housed for 1-2 weeks under controlled conditions (22–24°C, free access to food and water, natural day/night cycle) before initiation of the experiment. All experimental procedures were carried out with the approval of the Second Military Medical University Institutional Animal Care and Use Committee.

### 2.2. Methane-Rich Saline (MS) Production and Measurement

As described by Ye et al. [17], pure methane (>99.9%) stored in a gas canister (Shanghai Jiliang Standard Gas Co., Ltd., Shanghai, China) was dissolved in physiological saline for 3 h under 0.4 MPa to a supersaturated level. The supersaturated MS was stored under atmospheric pressure at 4°C and was freshly prepared 1 day before administration to the animals to ensure the methane level of injection. Gas chromatography (Gas Chromatography-9860; Qiyang Co., Shanghai, China) was implemented to disclose the content of methane in the blood and the saline solute 1 day after the preparation. According to calculation, the concentration of the MS was 0.99 mmol/l. In addition, the degassing of the supersaturated MS stored under atmospheric pressure at 4°C was inevitable and the level of methane in the blood remained effective.

### 2.3. Experimental Design

According to previous studies [18], ALI was induced in rats by intraperitoneal (i.p.) injection of lipopolysaccharide (LPS) (20 mg/kg *Escherichia coli B55:5*, Sigma Aldrich, USA) in physiological saline. Prior to the experiment, rats were restrained from eating and drinking for 8 h. 90 rats were equally randomized to 5 groups: (1) the blank group, where rats received i.p. administration of physiological saline (20 ml/kg) per 12 h; (2) the MS group, where rats received i.p. administration of MS (20 ml/kg) per 12 h; (3) the LPS group, where rats received i.p. administration of LPS, followed by i.p. infusion of physiological saline (20 ml/kg) per 12 h; (4) the 2 ml MS group, where rats received i.p. administration of LPS, followed by i.p. infusion of MS (2 ml/kg) per 12 h; and (5) the 20 ml MS group, where rats received i.p. administration of LPS, followed by i.p. infusion of MS (20 ml/kg) per 12 h. Saline or MS was injected at the beginning of LPS challenged.

### 2.4. Oxygenation Index Analysis

The oxygenation index and lung permeability index (LPI) were detected 24 h after LPS challenged or sham. Rats were anesthetized and given endotracheal intubation with a catheter. Then rats were ventilated mechanically with pure oxygen at 7 ml/kg (85 breaths/min). After 20 min ventilation, the arterial blood was obtained from the carotid artery and measured using a commercial blood gas analyzer (model ABL8000; Radiometer Copenhagen, Westlake, Ohio, USA). Oxygenation index equals PaO_2_/FiO_2_ ratio which is the ratio of arterial oxygen partial pressure to fractional inspired oxygen.

### 2.5. Determination of the Lung Wet-to-Dry (W/D) Ratio

The rats were euthanized with a lethal injection of chloral hydrate 24 h after LPS challenged. Then, the thorax was opened, and the left lung was harvested quickly, and the blood and liquid on the lung surface were hydrated by using the filter paper. The lung water content was measured by determining the W/D ratio. The left lung tissue was weighed immediately, and after being dried in an oven at 65°C for 48 h, the same left tissue was reweighed. The number of tissues in each group for W/D determination was 5 (*n* = 5).

### 2.6. Lung Histological Examination by Hematoxylin and Eosin (HE) Staining and Observation

The rats were euthanized with a lethal injection of chloral hydrate 24 h after LPS challenged and then perfused with 0.01 M PBS for 10 min firstly and then 4% paraformaldehyde (PFA) for 15 min. The harvested lung tissue was fixed in 4% PFA for 24 h, paraffin embedded, sliced to 5 mm sections, and stained with HE for observation under the light microscope (Olympus, Tokyo, Japan). The images were evaluated morphologically by scoring the histologic specimens by two pathologists in a blind test. Edema, hyperemia and congestion, neutrophil margination and tissue infiltration, intra-alveolar hemorrhage and debris, and cellular hyperplasia were scored as follows: absent (0), mild (1), moderate (2), and severe (3) [19]. A total score was calculated for each animal.

### 2.7. Transmission Electron Microscopy (TEM)

The lower lobes of the right lung were cut into 1-2 mm cubes and fixed in 2.5% glutaraldehyde at 4°C immediately in the sealed container, and a syringe was used to empty the gas in the lung tissue. After sinking to the bottom, the lung specimens were then treated with osmium tetroxide and dehydrated in serial concentrations of ethanol. The tissues were treated with propylene oxide again, and then with a propylene oxide/epoxy resin mixture. Finally, they were embedded in labeled capsules with freshly prepared resin by Epon812. Sections were stained with uranyl acetate and lead citrate and observed under a Hitachi-7650 TEM (Hitachi, Tokyo, Japan) at 10000 to 20000x magnifications.

### 2.8. Collection and Analysis of the Blood and Bronchoalveolar Lavage Fluid (BALF)

The rats were euthanized with an injection of chloral hydrate 24 h after LPS challenged. Inserting a number 7 needle into the tail vein and collecting the blood to test. The method for collecting BALF was as previously described [20]. The rat trachea was isolated by blunt dissection by inserting a blunt number 7 lumbar puncture needle into the airway and fixed with a wire. Five volumes of 5 ml physiological saline were instilled, gently aspirated, pooled, and reaspirated. There was no difference in the total volume of BALF recovered (21 ± 2.3 ml fluid) after the lung lavage process between the four groups, via centrifugation at 1500 g for 10 min at 4°C. The supernatant was collected and stored at −80°C. The cell types and total leukocyte count in the blood and BALF were determined using a hemocytometer (*n* = 8–10). The levels of tumor necrosis factor-*α* (TNF-*α*), interleukin-1*β* (IL-1*β*), and IL-6 in BALF were determined using rat ELISA kits (R&D Systems, Minneapolis, MN, USA) according to the manufacturer's instructions. The precipitated cells were resuspended by 0.01 M PBS, then centrifuged at 1500 g for 10 min. 10 *μ*l precipitated cells were smeared on a slide and stained by Giemsa. Images were captured with a light microscope to observe the cell type and shape. The concentration of proteins in BALF and plasma was analyzed via the bicinchoninic acid (BCA) method by using the BCA kit (Weiao, Shanghai, China) according to the manufacturer's instructions. The protein content was calculated by light density (OD) measured at 562 nm. The LPI was analyzed and calculated. LPI = concentration of proteins in BALF/concentration of proteins in plasma (×10^−3^).

### 2.9. Detection of Malondialdehyde (MDA) and Superoxide Dismutase (SOD) Activity Oxidative Assay

The marker of lipid oxidation level was measured by MDA activity assay, while the marker indicating the antioxidant level was measured by SOD activity assay. 24 h after LPS challenged, the rats were euthanized with an injection of chloral hydrate, and the right lower lobe lung samples were collected and homogenized in chilled PBS. Then, 100 *μ*g lung tissue was collected and homogenized immediately in 1 ml PBS at 4°C. The homogenate was centrifuged at 3000 g for 15 min at 4°C. The supernatant was collected and used to detect the level of MDA and SOD. The SOD activity (U/m) and MDA concentration (nmol/mg protein) were then measured using assay kits (Nanjing Jiancheng Bioengineering Institute, Nanjing, China). The absorbance was measured by spectrometry at 532 and 550 nm for MDA and SOD, respectively. The level of MDA and SOD was calculated by each absorbance (*n* = 8–10).

### 2.10. Detection of Apoptotic Cells by Terminal Deoxynucleotidyl Transferase dUTP Nick-End Labeling (TUNEL) Staining

24 h after LPS challenged, the ratio of apoptotic cells in lung paraffin-embedded sections was measured by TUNEL staining using the detection kit (Roche Molecular Biochemicals, Indianapolis, IN, USA) according to the manufacturer's instructions. Cells with brown-stained nuclei were considered apoptotic cells. The ratio of apoptotic cells was the percentage of TUNEL-positive cells and the total number of cells by using Image-Pro Plus 5.0 (Media Cybernetics, Silver Spring, USA). Ten random fields per section were analyzed and two pathologists performed the blind examination (*n* = 5).

### 2.11. Western Blotting

The expression of caspase-3 in the lung tissue was detected by western blot analysis using antibodies purchased from Cell Signaling Technology (CST #9662, Danfoss, MA, USA). 24 h after LPS challenged, the right upper lobe lung samples were collected and homogenized in tissue lysate. The total protein content in each sample was determined by the BCA method. Cytosolic fractions were separated by SDS-PAGE, transferred, and immobilized on a nitrocellulose membrane. The membrane was blocked by incubation with 5% nonfat dried milk in PBS for 2 h at room temperature and then incubated with anticaspase-3. Then, the membrane was washed in PBS with Tween 20 (PBST) 3 times. Using antirabbit horseradish peroxidase secondary antibody (1 : 15,000; Abmart, Shanghai, China) for 2 h at room temperature, the immune complexes were detected with the ECL chemiluminescence system. The levels of caspase-3 from densitometry were normalized to GAPDH level.

### 2.12. Survival Experiments

The survival experiments were performed in five groups of 75 rats (blank/MS/LPS/2 ml MS/20 ml MS, *n* = 15). While the LPS/2 ml MS/20 ml MS group was injected with LPS (20 mg/kg), the blank and MS groups were injected with the same weight physiological saline. The MS/2 ml MS/20 ml MS group was injected with MS at the same time. The survival of the rats was monitored for 96 h after LPS challenged.

### 2.13. Statistical Analysis

The SPSS 21.0 software (SPSS Inc., Chicago, USA) was used for all statistical analyses. The figures were treated by using Prism version 6.0 software (GraphPad Inc., CA, USA). The data are reported as means ± SD, while *n* is the number of animals in the study group. One-way ANOVA or Student's *t*-tests were applied as well as regression analysis. Comparisons between survival rates of different groups were made with the Kaplan-Meier and Mantel-Cox methods. Values of *P* < 0.05 were regarded as statistically significant.

## 3. Results

### 3.1. Effect of MS on the Survival Rate of LPS-Induced ALI Rats

**Figure 1:**
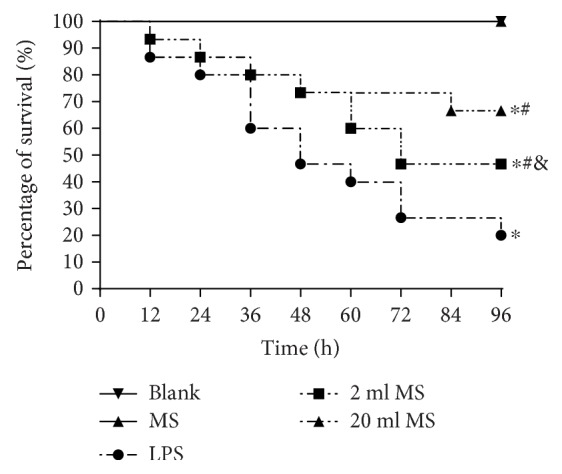
MS treatment improved the survival rate of LPS-induced ALI rats. The rats were challenged by LPS (20 mg/kg) with or without MS injection. The survival rate was observed 96 h after exposure to LPS. Survival was analyzed by log-rank (Mantel-Cox) test (*n* = 15), ^∗^*P* < 0.05 versus that in the blank group; ^#^*P* < 0.05 versus that in the LPS group; ^&^*P* < 0.05 versus that in the 2 ml MS group.

At 3 h after LPS challenged, rats manifested lethargy, piloerection, diarrhea, huddling, malaise, and apparent signs of endotoxic shock. As shown in Figure 1, there was no significant difference between the blank and MS groups, indicating that the MS had no significant effect on the rat. The survival rate in the LPS, 2 ml MS, and 20 ml MS groups was significantly lower than that in the blank and MS groups, indicating that the survival rate was decreased significantly in LPS-challenged rats. The survival rate in the 20 ml MS group was significantly higher than that in the other four groups (*P* < 0.05, *n* = 15). This observation indicates that injection of MS could improve the survival rate of LPS-challenged ALI.

### 3.2. Effect of MS on the Pulmonary Function and Pulmonary Permeability

**Figure 2:**
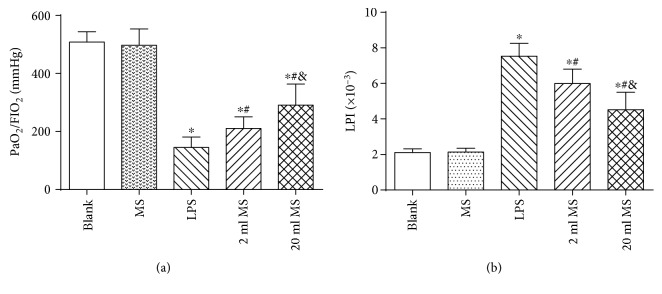
MS treatment improved the pulmonary function and pulmonary permeability of LPS-induced ALI rats. Data are presented as mean ± SD (*n* = 8–10). ^∗^*P* < 0.05 versus that in the blank group; ^#^*P* < 0.05 versus that in the LPS group; ^&^*P* < 0.05 versus that in the 2 ml MS group.

The rat pulmonary function was tested by the PaO2/FiO2 (Figure 2(a)), and the pulmonary permeability (Figure 2(b)) was evaluated by LPI at 24 h after LPS challenged. There was no significant difference in the PaO2/FiO2 and LPI between the blank and MS groups. The PaO2/FiO2 was significantly decreased, and LPI was significantly increased in LPS-challenged rats, which were markedly improved by MS injection treatment (*P* < 0.05, Figure 2). These results demonstrate that MS treatment significantly improves the pulmonary function and upregulate the pulmonary permeability in LPS-challenged rats. Additionally, the effect of methane was in a dose-dependent manner.

### 3.3. Effect of MS on the Lung W/D Ratio

**Figure 3:**
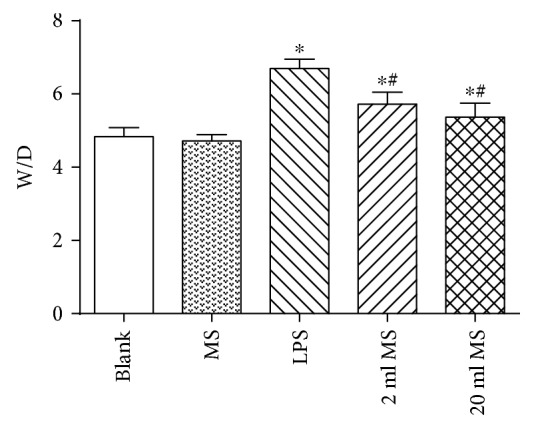
MS treatment decreased the lung W/D ratio of LPS-induced ALI rats. Data are presented as mean ± SD (*n* = 5). ^∗^*P* < 0.05 versus that in the blank group; ^#^*P* < 0.05 versus that in the LPS group.

At 24 h after LPS challenged, the left lung W/D ratio was calculated to detect lung injury in rats. As shown in Figure 3, there was no significant difference in the W/D ratio between the blank and MS groups (*P* > 0.1). The lung W/D ratio in the LPS, 2 ml MS, and 20 ml MS groups was significantly higher than that in the blank and MS groups (*P* < 0.05). The W/D ratio in the 2 ml MS and 20 ml MS groups was significantly lower than that in the LPS group (*P* < 0.05), indicating that LPS challenged could significantly increase the W/D ratio. Methane treatment could significantly decrease the W/D ratio of LPS-challenged rats, but had no significant effect on the normal rats.

### 3.4. Histological Examination and Ultrastructural Organization of Lung Tissues

**Figure 4:**
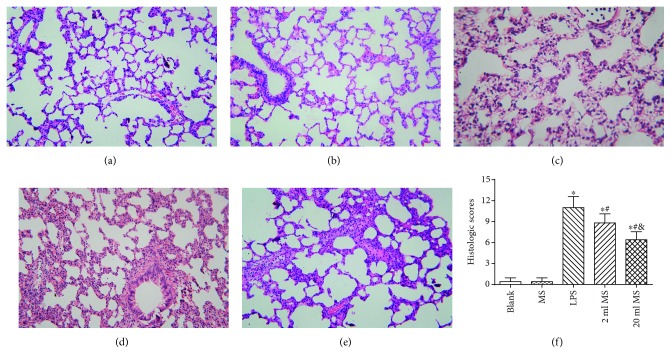
Histologic assessment of MS treatment attenuated ALI in rats using HE staining (200x). (a) Blank group; (b) MS group; (c) LPS group; (d) 2 ml MS group; (e) 20 ml MS group; (f) lung histological scores. The values are expressed as mean ± SD (*n* = 8–10). ^∗^*P* < 0.05 versus that in the blank group; ^#^*P* < 0.05 versus that in the LPS group; ^&^*P* < 0.05 versus that in the 2 ml MS group.

**Figure 5:**
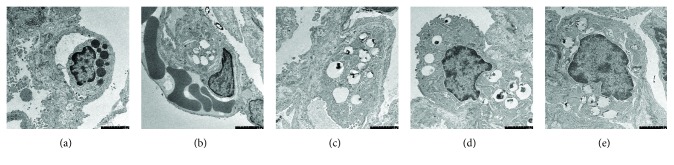
Lung ultrastructural histopathologic change of MS treatment attenuated ALI in rats. (a) Blank group; (b) MS group; (c) LPS group; (d) 2 ml MS group; (e) 20 ml MS group.

There was no significant difference in histological examination and ultrastructural organization of lung tissues between the blank and MS groups. The macroscopic observation showed pink, smooth, and glossy lung surfaces in the blank and MS groups, without hemorrhage and infiltration. With respect to histopathologic changes, light microscopic observation showed that the constitution of the lung was typical, with thin alveolar walls, with no neutrophil infiltration in the lung interstitial and alveolar spaces (Figures 4(a) and 4(b)). With respect to ultrastructural histopathologic changes by electron microscopy, the alveolar epithelial cells showed the typical constitution of mitochondria and endoplasmic reticula (Figures 5(a) and 5(b)). LPS challenged induced lung injury characterized by alveolar wall thickening, neutrophil infiltration in the lung interstitial and alveolar spaces, consolidation, and alveolar hemorrhage, which were present in the LPS (Figure 4(c)), 2 ml MS (Figure 4(d)), and 20 ml MS groups (Figure 4(e)). MS treatment reduced the number of infiltrated inflammatory cells and improved the lung architecture markedly as compared with the LPS group. There was no significant difference in lung histological scores between the blank and MS groups (Figure 4(f), *P* > 0.1). The scores in LPS, 2 ml MS, and 20 ml MS were increased significantly as compared with those in the blank and MS groups (*P* < 0.05), while the score in the 20 ml MS group was significantly lower than that in the LPS group (*P* < 0.05) (Figure 4(f)). With respect to ultrastructural histopathologic changes by electron microscopy, Type I alveolar cells were characterized by vacuolation, degranulation, mitochondrial swelling, perinuclear cisterna dilation, and rough endoplasmic reticulum pool after inhalation injury, while Type II alveolar epithelial cells were typically characterized by vacuolation of lamellar bodies and exfoliated tubular myelin. The alveolar structure was improved apparently after MS treatment when compared with that in the LPS group (Figure 5).

### 3.5. Analysis of the Blood and BLAF

**Figure 6:**
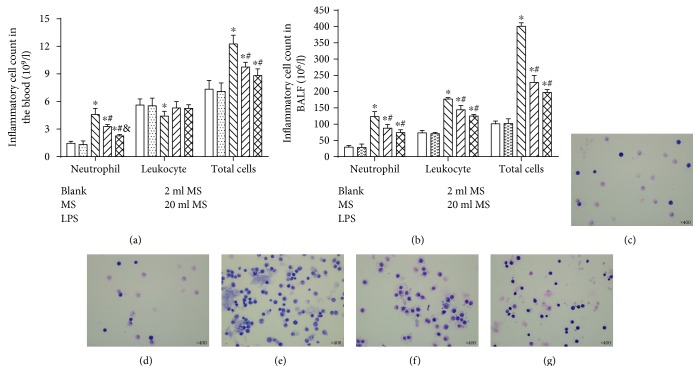
MS treatment decreased the number of neutrophils and lymphocytes in the blood and BALF in LPS-induced ALI rats. (a) The number of lymphocytes and the total number of neutrophils in the blood; (b) the number of lymphocytes and the total number of neutrophils in BLAF; (c–g) the type and shape of inflammatory cells in BALF by Giemsa staining (400x). Data are presented as mean ± SD (*n* = 8–10). ^∗^*P* < 0.05 versus that in the blank group; ^#^*P* < 0.05 versus that in the LPS group.

Neutrophilic granulocyte and lymphocyte was analyzed in the blood (Figure 6(a)) and BALF (Figure 6(b)) by hemocytometer. There was no significant difference in the total number and cell type between the blank and MS groups (*P* > 0.1). Methane treatment had no effect on the number and type of the cells in the normal rats. The neutrophil cell number in the LPS, 2 ml MS, and 20 ml MS groups was significantly greater than that in the blank and MS group in the blood and BALF (*P* < 0.05). LPS challenged significantly increased the total number of cells, especially neutrophil and lymphocytes in BALF (*P* < 0.05). Methane treatment had no effect on the number of leukocyte in the blood 0 (*P* > 0.1). The type and shape of inflammatory cells in BALF by Giemsa staining (Figures 6(c)–6(g)). MS treatment significantly reduced the total number and the type of neutrophil and lymphocytes in the blood and BALF.

### 3.6. The Effect of MS Treatment on Lung TNF-*α*, IL-6, and IL-1*β* in BALF of LPS-Challenged Rats

**Figure 7:**
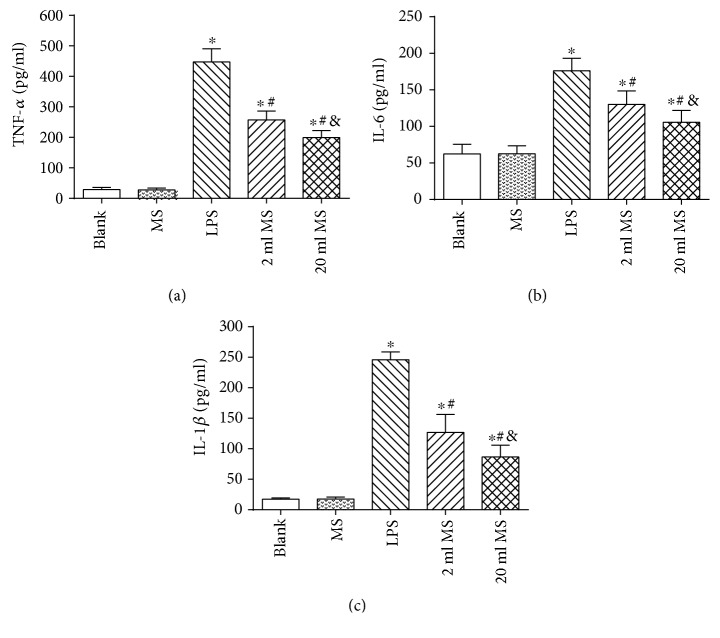
The expression of TNF-*α*, IL-6, and IL-1*β* in BALF. Data are reported as means ± SD (*n* = 8–10). ^∗^*P* < 0.05 versus that in the blank group; ^#^*P* < 0.05 versus that in the LPS group; ^&^*P* < 0.05 versus that in the 2 ml MS group.

At 24 h after LPS challenged, the levels of lung TNF-*α*, IL-6, and IL-1*β* in BALF were analyzed (*n* = 8–10). As shown in Figure 7, TNF-*α*, IL-6, and IL-1*β* levels in the LPS, 2 ml MS, and 20 ml MS groups were significantly higher than those in the blank and MS groups (*P* < 0.05). There was no significant difference between the blank and MS groups (*P* > 0.1). TNF-*α*, IL-6, and IL-1*β* levels in the 2 ml MS and 20 ml MS groups were significantly lower than those in the LPS group (*P* < 0.05). LPS challenged significantly increased the expression of TNF-*α*, IL-6, and IL-1*β*. Methane treatment significantly decreased their expressions in LPS-challenged rats (*P* < 0.05) but had no significant effect on the normal rats (*P* > 0.05).

### 3.7. Antioxidative Effect of MS Treatment on the Lung Challenged by LPS

**Figure 8:**
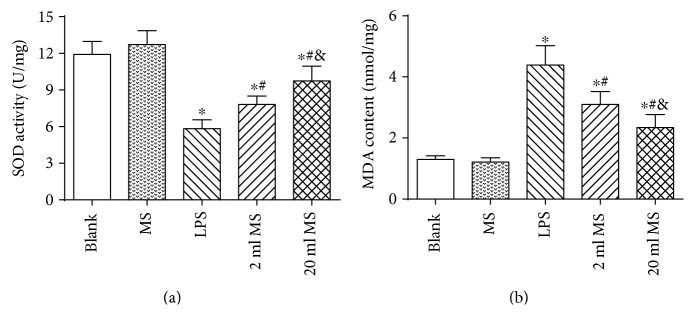
Methane treatment upregulated the activity of lung antioxidant enzyme SOD and reduced the level of lung oxidative product MDA. Data are presented as mean ± SD (*n* = 8–10). ^∗^*P* < 0.05 versus that in the blank group; ^#^*P* < 0.05 versus that in the LPS group; ^&^*P* < 0.05 versus that in the 2 ml MS group.

To explore the potential mechanism underlying the protective effect of MS on the lung challenged by LPS, MDA and SOD levels were measured, knowing that they are both regular representatives for oxidant-induced injury. As shown in Figure 8, the expression of MDA was significantly increased and the expression of SOD was significantly decreased in the LPS, 2 ml MS, and 20 ml MS groups as compared with that in the blank and MS groups (*P* < 0.05). However, there was no significant difference between the blank and MS groups (*P* > 0.1). The SOD activity was increased and the MDA activity was decreased significantly in the 20 ml MS group as compared with that in the LPS group (*P* < 0.01). The antioxidative effect of methane on the lung challenged by LPS was in a dose-dependent manner.

### 3.8. Antiapoptotic Effect of MS Treatment on the Lung Challenged by LPS

**Figure 9:**
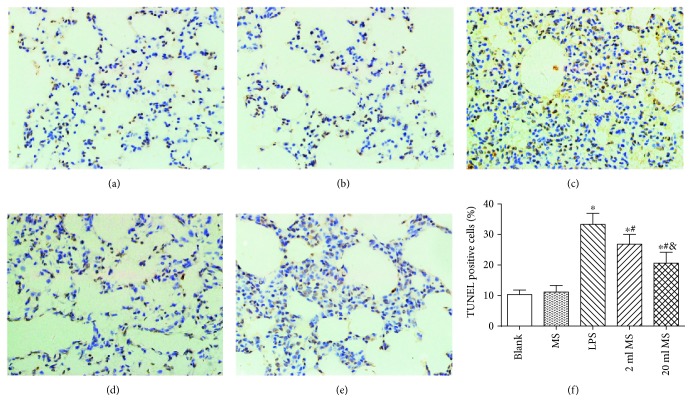
Antiapoptotic effect of MS treatment on ALI by using the TUNEL staining (400x). (a) Blank group; (b) MS group; (c) LPS group; (d) 2 ml MS group; (e) 20 ml MS group; (f) AI TUNEL-positive cell count of lung cells. Data are presented as mean ± SD (*n* = 5). ^∗^*P* < 0.05 versus that in the blank group; ^#^*P* < 0.05 versus that in the LPS group; ^&^*P* < 0.05 versus that in the 2 ml MS group.

**Figure 10:**
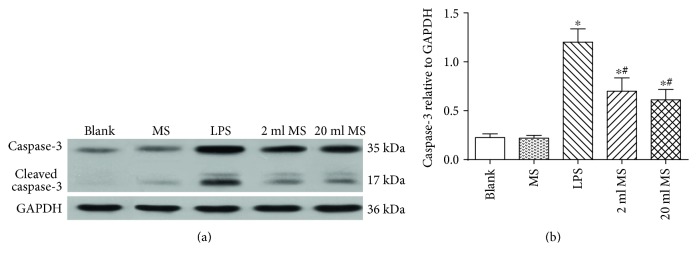
Antiapoptotic effect of MS on ALI by western blotting the protein expression of caspase-3. Data are presented as mean ± SD (*n* = 5). ^∗^*P* < 0.05 versus that in the blank group; ^#^*P* < 0.05 versus that in the LPS group.

The apoptosis of the lung tissue was detected by TUNEL staining (Figure 9), and caspase-3 content was tested by western blot to see how methane affected the apoptotic process (Figure 10). TUNEL staining showed that the nuclei of normal cells were stained blue, while the nuclei of apoptotic cells were stained brown. A few cells were stained brown in the blank and MS groups, while large numbers of positive cells were stained brown in the LPS, 2 ml MS, and 20 ml MS groups, showing a significant difference (Figure 9(f), *P* < 0.05). Positive cells in the 20 ml MS group were significantly reduced compared with those in the LPS group (*P* < 0.05). The statistical result of the apoptotic index (AI) for each group was also consistent with the above statement (Figure 9(f)). Western blotting the protein of caspase-3 showed the same trend of AI. The gray-scale ratio of the caspase-3 in the 2 ml MS and 20 ml MS groups was reduced significantly compared with that in the LPS group (*P* < 0.05) (Figure 10). The trend of apoptosis showed by TUNEL staining and western blotting of caspase-3 indicated that methane treatment prevented lung cell apoptosis in rats with inhalation injury.

## 4. Discussion

Methane as the most abundant and simplest alkane organic gas in the atmosphere is important for the greenhouse effect. Bacteria in the human gut can produce methane and hydrogen gas, while hydrogen can be converted to methane in the gut by methane-producing bacteria [11, 12]. However, the physiological role of methane in the human body has long been overlooked. Recently, hydrogen has been demonstrated to have antioxidative, anti-inflammatory, and antiapoptotic effects on animal lung ALI [8, 21, 22]. However, there is little knowledge about the function of methane in disease treatment. Some studies reported that methane had positive effects on diabetic retinopathy [23], animal intestinal IR injury [24], and liver IRI [17] via its antioxidative, anti-inflammatory, and antiapoptotic activities [25]. It was first demonstrated in our study that i.p. administration of MS had positive protective effects against LPS-induced ALI via its antioxidative, anti-inflammatory, and antiapoptotic activities. The i.p. injection of MS could significantly improve the survival rate of LPS-induced ALI rats (Figure 1) and the pulmonary function (Figure 2), reduce the content of inflammatory cytokines in BALF, and relieve pulmonary inflammation induced by LPS (Figure 7). In addition, MS could significantly increase the content of SOD, decrease the content of MAD and other products of oxidative stress, and reduce tissue oxidative stress injury (Figure 8). In terms of apoptosis of the lung tissue, methane-rich water inhibited the expression and activation of caspase-3 in the lung tissue and attenuated lung damage by decreasing the ratio of cell apoptosis (Figures 9 and 10).

ARDS characterized by progressive dyspnea and refractory hypoxemia is caused by a variety of inflammatory mediators, effector cells activated, cascade amplifying inflammation, and a secondary diffuse lung parenchyma damaged, which are relatively common clinical syndromes associated with high morbidity and mortality, especially in ICU patients [26]. Sepsis is a common and critical clinical entity and the major cause of ALI [27]. Sepsis can cause damage to all organs in the body. The lung is the most susceptible organ to be affected in the progression of ALI. LPS is the main component of the Gram-negative bacterial cell wall and implicated as an important toxin that precipitates lung injury. Knowing that there is a significant correlation between the severity of ALI and the extent of LPS, we used LPS (20 mg/kg) to establish animal models of ALI by i.p. injection in rats [28]. It was found that LPS induced tachypnea, less activity, diarrhea, and other symptoms associated with lung damage and dysfunction in rats (Figure 4). The LPS-induced lung tissue stained by HE was characterized by alveolar wall thickening, neutrophil infiltration in the lung interstitial and alveolar space, consolidation, and alveolar hemorrhage. At the same time, the lung W/D ratio, content of inflammatory cytokines, and the amount of cells in BALF were increased significantly, which is consistent with previous studies [29]. In this study, we demonstrated that MS could significantly increase the survival rate (Figure 1) and reduce leakage of the lung tissue and W/D ratio in rats (Figure 3).

Recent evidence [30] suggests that the main pathogenesis of ALI characterized by progressive dyspnea and refractory hypoxemia is associated with the imbalance of cytokines, oxidative stress, and apoptosis, causing extensive pulmonary vascular injury. Therefore, the antioxidative, anti-inflammatory, and antiapoptotic activities are considered to be an effective way to protect ALI. The imbalance of pro- and anti-inflammatory cytokine is considered to be the most important mechanism in ALI. Various inflammatory mediators are known to be involved in ALI. Ample evidence indicates that proinflammatory cytokines, notably TNF-*α*, IL-1*β*, and IL-6, participate in the early development of inflammation [31]. While neutrophils and lymphocytes contacted with these pathogeneses, they have been demonstrated to play crucial roles in ALI [32]. The retention and aggregation of a large number of neutrophils can be seen in the ALI lung tissue. On the one hand, the retention of neutrophils in the pulmonary capillary bed could lead to pulmonary microcirculation for mechanical obstruction, and the neutrophils could release a large number of inflammatory factors and oxidative stress which increased the lung injury [33]. LPS can induce the redistribution of lymphocytes from the peripheral blood and spleen to the lymphoid tissue [34]. The effect of LPS on lymphocytes in the peripheral blood was smaller than that of neutrophils, and the change range was smaller and lasts longer [35]. Based on this understanding, we postulated that the content of inflammatory cytokines in BALF and the morphologic and number changes of leukocyte in BALF and the blood could be used to determine the intensity of inflammatory responses in the lung tissue accurately (Figures 6 and 7). In this study, we detected the morphologic and number changes of leukocyte in BALF and the blood and the changes in the content of inflammatory cytokines in BALF. We found that MS could significantly reduce the systemic inflammation especially in the lung tissue after LPS-induced ALI.

A growing number of studies have found that excessive production of ROS and reduction of antioxidant defense systems play important roles in the pathogenesis of LPS-induced ALI [36]. The balance of oxidation and antioxidation mechanisms exists in vivo under the physiological conditions. However, the imbalance due to neutrophil activation and accumulation in the lung may produce large amounts of ROS and their by-products, thus weakening the antioxidant system and causing oxidative damage. Because of the excessive production of ROS and their by-products, direct damage of the cell membrane unsaturated fatty acids decreases the capability of liquidity and increases permeability of the membrane, and on the other hand, ROS are released into the lung tissue, causing direct damage to alveolar epithelial cells and pulmonary vascular endothelial cells. The blood barrier integrity is destroyed and the permeability is increased, leading to pulmonary edema. ROS and their by-products can activate the expression of NF-*κ*B [37], which is known to play the most crucial role in cell apoptosis. A previous study showed that pretreatment with free radical scavengers could effectively reduce the release of inflammatory cytokines in BALF and reduce the extent of lung damage in the LPS-induced ALI mouse model. Therefore, seeking a method to reduce oxidative stress in ALI is an important potential strategy for the treatment of ALI. In this experiment, we used the content of MDA and SOD in the lung tissue as an indicator to reflect the extent of oxidative stress and lung tissue injury (Figure 9). MDA is produced from the cell membrane and mitochondria film-occurred lipid peroxidation, which reflects the activity and amount of ROS in vivo directly and the degree of cell injury indirectly [38]. SOD is an important antioxidant enzyme that can eliminate superoxide anion and reduce damage caused by the form of prototype nitrite and has a protective effect on the lung. There is a significant negative correlation between the expression level of SOD and the extent of lung injury [39], while the products of oxidative stress can reduce the activity of SOD.

The Fas signaling pathway is activated in ALI. The Fas/FasL system is an important pathway of apoptosis mediated by caspase [40]. Fas, the receptor of death signals, initiates the pathway of apoptosis mediated by caspase-3. The activation of Fas can induce the apoptosis of Type II alveolar epithelial cells and activate monocytes and macrophages at the same time. The inflammatory factors released by the activated monocytes and macrophages can enhance the sensitivity of Fas-induced apoptosis in alveolar epithelial cells [41]. The pathological process of ALI is the process of lung cell apoptosis. However, the association between cell apoptosis and inflammation remains unclear. In this study, TUNEL assay was used to detect lung cell apoptosis at the organizational level. The expression of the caspase-3 protein was measured by western blot (Figures 9 and 10). It was found that the apoptosis rate of the lung tissue was increased significantly in ALI, and methane-rich water could significantly reduce the expression of the cleaved caspase-3 and decrease the ratio of apoptosis.

However, the exact mechanism underlying the antioxidative, anti-inflammatory, and antiapoptotic activities of methane remains unclear. Different researchers have proposed different hypotheses. Boros et al. [14] hypothesized that methane might accumulate transiently at the interfaces of cell membranes, thereby changing the physicochemical properties or the in situ functionality of proteins embedded within this environment. Kai et al. [42] hypothesized that methane might exert effects on membrane channels, affect G-proteins, membrane or receptor-mediated signaling, and acetylcholine-activated ion channel kinetics. Fink et al. [43] proposed that more studies should be performed to elucidate whether mammalian cells contain an oxygenase that is capable of using methane as a substrate, whether the biological effects of methane are caused by the formation of small amounts of the reactive alcohol, methanol, and/or changes in the redox milieu of the cell due to changes in NAD(P)+/NAD(P)H ratio, and whether there is a cellular “receptor” for methane. In addition, because there is no study about the comparison of the effects of methane intraperitoneal administration as compared to that of inhalation, we adopted the simpler, controllable, and safer way of intraperitoneal administration in this study, but the therapeutic effects comparison on ALI between different modes of medication should be further researched. Several problems should be resolved before methane could be widely applied to clinical practice [16].

## 5. Conclusion

The present study has demonstrated that MS exerts a protective effect against LPS-induced ALI in rats through its antioxidative, anti-inflammatory, and antiapoptotic activities in a dose-dependent manner. Although the etiology and mechanisms of methane protecting ALI/ARDS have not been clarified, methane is easy to obtain, relatively stable and atoxic, and can permeate cell membranes with few adverse effects. Therefore, methane might be a promising gas for the clinical treatment of ALI. The timing, optimal way, and exact mechanism of methane administration for the clinical treatment of ALI need to be explored in future studies.
